# Distribution of Copper in Rats Submitted to Treatment With Copper Aspirinate

**DOI:** 10.1155/MBD.1998.333

**Published:** 1998

**Authors:** Weiping Liu, Yikun Yang, Huizhou Xiong, Ying Lu, Rong Yang

**Affiliations:** 1 Institute of Precious Metals Kunming 650221 China; 2 Kunming Medical College Kunming 650031 China

## Abstract

The distribution of copper in Sprague – Dawley rats following a three month oral administration of 0,10 or 50mg/kg copper aspirinate has been investigated. Metal content was determined by ICP – AES in blood, brain, kidney, liver, lung, spleen, and dejection. The results show that treatment with copper aspirinate did not cause accumulation of copper in rats and excess ingested copper was excreted through feces.


### DISTRIBUTION OF COPPER IN RATS SUBMITTED TO TREATMENT WITH COPPER ASPIRINATE

Weiping Liu*[1], Yikun Yang[1], Huizhou Xiong[1], Ying Lu[2], and Rong Yang[2]

[1] Institute of Precious Metals, Kunming,650221, P. R. China
[2] Kunming Medical College, Kunming,650031, P. R. China

**Abstract**

The distribution of copper in Sprague−Dawley rats following a three month oral administration of 0,10 or 50mg/kg copper aspirinate has been investigated. Metal content was determined by ICP−AES in blood,brain,kidney,liver,lung,spleen, and dejection. The results show that treatment with copper aspirinate did not cause accumulation of copper in rats and excess ingested copper was excreted through feces.

### 1. Introduction

Since the first report by Prof. J. R. J. Sorenson that copper complexes of many ligands had anti−inflammatory activity[1],copper aspirinate[$Cu_2(ASA)_4$],the copper complex of aspirin, has been found to be much more effective as an anti−thrombotic agent[2] as well as an anti−inflammatory agent[3].In addition,it has antiulcer activity[4] which distinguishes it from aspirin. It is,therefore,suggested that copper aspirinate should be clinically used in treating thrombotic and arthritic diseases[2][3]. However doctors are still concerned about the relative large amount of copper that may be ingested. Excess copper intake may be harmful to mankind although it is an essential metalloelement. Prof. J. R. J. Sorenson has studied the preliminary toxicity of copper aspirinate[5]. As an expansion of this toxicity study and part of the pharmacological evaluation,the present investigation is made to see whether accumulation of copper will occur in rats following a three month oral treatment with 0,10 or 50mg/kg copper aspirinate.

### 2. Materials and Methods

Copper aspirinate was prepared and purified by the method previosly described[1]. Elemental analysis, IR, UV and MS−$FAB^+$ corresponded to the known chemical structure[6]. The complex was suspended in 1.4% polyvinyl alcohol and 5% propylene glycol immediately prior to intragastric administration.

Three groups of twenty Sprague−Dawley rats of either sex were given orally the vehicle,10,or 50 mg/kg copper aspirinate six days a week for three months. Treatment ceased at the end of the third month. The remaining animals were continued without treatment for an additional month. These rats were massed weekly during treatment and doses of copper aspirinate changed weekly according to body mass in order to maintain the original doses of 10 or 50 mg/kg.

At the end of each month,five rats were killed by cervical dislocation.Blood samples were collected immediately by cardiac puncture. Samples of liver,kidney,spleen and brain were dissected and massed. Feces were also collected at the end of the third month. All samples were frozen and stored prior to metal content analysis. After thawing,samples were digested in concentrated nitric acid at 90℃ ,and then perchloric acid was added,followed by a gentle heating. The resulting solutions were clear. After diluted,they were analyzed for copper content using Inductively Coupled Argon Atomic Emission Spectrophotometry (ICP－AES) at 324.7nm.

Table 1. Blood copper concentration ($x \pm$ S.E.M , $n=5$) for non－treated and copper aspirinate－treated rats at the end of each month

| Group | Dose | Cu(μg/g wet mass) | | | |
|---|---|---|---|---|---|
| | | 30 days | 60 days | 90 days | 120 days |
| Control | | 1.05 ± 0.20 | 1.10 ± 0.10 | 1.30 ± 0.20 | 1.24 ± 0.10 |
| $Cu_2(ASA)_4$ | 10 mg/kg | 1.12 ± 0.30 | 1.14 ± 0.10 | 1.16 ± 0.30 | 1.16 ± 0.20 |
| | 50 mg/kg | 1.20 ± 0.20 | 1.31 ± 0.20 | 1.26 ± 0.20 | 1.18 ± 0.20 |

Table 2. Liver copper concentration ($x \pm$ S.E.M, $n=5$) for non－treated and copper aspirinate－treated rats at the end of each month

| Group | Dose | Cu(μg/g wet mass) | | | |
|---|---|---|---|---|---|
| | | 30 days | 60 days | 90 days | 120 days |
| Control | | 3.10 ± 0.20 | 4.10 ± 0.50 | 4.40 ± 0.50 | 4.30 ± 0.50 |
| $Cu_2(ASA)_4$ | 10 mg/kg | 3.55 ± 0.40 | 4.20 ± 0.40 | 4.30 ± 0.50 | 3.80 ± 0.30 |
| | 50 mg/kg | 3.30 ± 0.50 | 4.30 ± 0.40 | 4.20 ± 0.60 | 4.10 ± 0.60 |

### 3. Results and Discussion

Tissue distribution of copper are shown in Table 1 to 5. There were no significant differences in copper concentration among non－treated,10 mg/kg and 50 mg/kg copper aspirinate－treated groups. This means chronic oral treatment with 10 or 50mg/kg copper aspirinate did not cause accumulation of copper in blood,liver,kidney,brain or spleen. The 50 mg/kg dose of copper aspirinate represents a daily intake of 7.5 mg copper per kg,much larger than needed for normal metabolism,but did not cause accumulation. These results are consistent with the understanding that normal animals can excrete ingested copper in excess of homeostatic requirements.

Table 3. Kidney copper concentration ($x \pm$ S.E.M, $n=5$) for non－treated and copper aspirinate－treated rats at the end of each month

| Group | Dose | Cu(μg/g wet mass) | | | |
|---|---|---|---|---|---|
| | | 30 days | 60 days | 90 days | 120 days |
| Control | | 5.60 ± 1.30 | 5.70 ± 1.30 | 5.90 ± 1.20 | 6.20 ± 1.20 |
| $Cu_2(ASA)_4$ | 10 mg/kg | 4.80 ± 1.10 | 5.50 ± 1.00 | 5.70 ± 1.40 | 6.50 ± 1.30 |
| | 50 mg/kg | 5.40 ± 0.90 | 6.40 ± 2.10 | 6.10 ± 1.50 | 5.80 ± 1.10 |

Table 4. Spleen copper concentration (x ± S.E.M, n = 5) for non treated and copper aspirinate – treated rats at the end of each month

| Group | Dose | Cu(μg/g wet mass) | | | |
|---|---|---|---|---|---|
| | | 30 days | 60 days | 90 days | 120 days |
| Control | | 1.10 ± 0.80 | 1.30 ± 0.30 | 1.40 ± 0.20 | 1.30 ± 0.20 |
| $Cu_2(ASA)_4$ | 10 mg/kg | 1.40 ± 0.30 | 0.90 ± 0.10 | 1.10 ± 0.30 | 1.40 ± 0.10 |
| | 50 mg/kg | 1.50 ± 0.50 | 1.40 ± 0.30 | 1.60 ± 0.40 | 1.50 ± 0.30 |

Data presented in Table 6 show that copper concentration in feces of treated animals, particularly with a dose of 50 mg/kg, was much higher than that of non – treated animals,which suggests that excess copper intake was excreted via feces. Bile may serve as the major excretory vehicle.

Table 5. Brain copper concentration (x ± S.E.M, n = 5) for non – treated and copper aspirinate – treated rats at the end of each month

| Group | Dose | Cu(μg/g wet mass) | | | |
|---|---|---|---|---|---|
| | | 30 days | 60 days | 90 days | 120 days |
| Control | | 2.20 ± 0.20 | 2.40 ± 0.20 | 2.10 ± 0.30 | 2.60 ± 0.40 |
| $Cu_2(ASA)_4$ | 10 mg/kg | 2.10 ± 0.10 | 2.60 ± 0.40 | 2.50 ± 0.20 | 2.20 ± 0.30 |
| | 50 mg/kg | 2.50 ± 0.30 | 2.80 ± 0.50 | 2.30 ± 0.10 | 2.40 ± 0.20 |

Table 6. Copper concentration(x ± S.E.M, n = 20) in feces for non – treated and copper aspirinate – treated rats at the end of the third month

| Group | Dose | Cu(μg/g wet mass) |
|---|---|---|
| control | | 38.00 ± 3.00 |
| $Cu_2(ASA)_4$ | 10 mg/kg | 184.00 ± 5.00 |
| | 50 mg/kg | 496.00 ± 10.00 |

The projected human dose of copper aspirinate is 2 mg/kg daily with a decrease to some maintenance dose with remission[7]. The 50 mg/kg dose used in these studies is 25 times the projected therapeutic dose but does not cause accumulation of copper. We conclude that copper aspirinate is safe for clinical use.

### References

1. J.R.J.Sorenson,*J.Med.Chem.*,1976,19,135
2. Shen Zhiqiang,Liu Weiping,*Acta Pharmacological Sinica*,1997,18(3),358
3. J.R.J.Sorenson,*Prog.Med.Chem.*,1989,26,437
4. L.J.Hayden,C.Thomas and G.B.West,*J.Pharm.Pharmacol*,1978,30,244
5. J.R.J.Sorenson,T.M.Rolniak and L.W.Chang,*Inorg.Chim.Acta*,1984,91,L31 – 34
6. Liu Weiping,Li Ling,*Academic J.Kunming Med.College*,1996,17(1),1
7. F.Kolbrunner,*German Patent*,DE 3033354(1982)

**Received: June 22, 1998 - Rejected: July 1, 1998 -**
**Accepted in revised camera-ready format: November 18, 1998**

